# Dr. Alfred Day

**Published:** 1909-04

**Authors:** 


Dr. Alfred Day, of Buffalo, died at his home in this city February 12, 1909, aged 75 years. He graduated at the University of Buffalo in 1887.

